# Prevalence and extent of heteroresistance by next generation sequencing of multidrug-resistant tuberculosis

**DOI:** 10.1371/journal.pone.0176522

**Published:** 2017-05-18

**Authors:** Darwin J. Operario, Alexander F. Koeppel, Stephen D. Turner, Yongde Bao, Suporn Pholwat, Sayera Banu, Suporn Foongladda, Stellah Mpagama, Jean Gratz, Oleg Ogarkov, Svetlana Zhadova, Scott K. Heysell, Eric R. Houpt

**Affiliations:** 1 Division of Infectious Diseases and International Health, Department of Medicine, University of Virginia, Charlottesville, VA, United States of America; 2 Department of Public Health Sciences, University of Virginia, Charlottesville, VA, United States of America; 3 Department of Microbiology, University of Virginia, Charlottesville, VA, United States of America; 4 International Centre for Diarrhoeal Disease Research, Bangladesh, Dhaka, Bangladesh; 5 Siriraj Hospital, Mahidol University, Bangkok, Thailand; 6 Kibong’oto Infectious Diseases Hospital, Kilimanjaro, Tanzania; 7 Scientific Centre for Family Health and Human Reproduction Problems, Irkutsk, Siberia, Russian Federation; St Petersburg Pasteur Institute, RUSSIAN FEDERATION

## Abstract

Amplicon-based Next Generation Sequencing (NGS) is an emerging method for *Mycobacterium tuberculosis* drug susceptibility testing (DST) but has not been well described. We examined 158 clinical multidrug-resistant *M*. *tuberculosis* isolates via NGS of 11 resistance-associated gene regions covering 3519 nucleotides. Across these gene regions, complete resistance or heteroresistance (defined as 1%-99% mutation) was present in at least one isolate in 6.3% of loci. The number of isolates with heteroresistance was highest for *gyrA* codon 94, *rpoB* codons 526 and 531, and *embB* codons 306, 372 and 406 (range 11–26% of isolates exhibited heteroresistance). 57% of MDR strains had heteroresistance of one or more recognized resistance-associated mutation. Heteroresistant loci generally exhibited high or low degrees of mutation (>90% or <10%). The deep sensitivity of NGS for detecting low level *pncA* heteroresistance appeared to improve genotypic-phenotypic PZA susceptibility correlations over that of Sanger. NGS demonstrates that heteroresistance in TB in the regions of key genes is common and will need to be bioinformatically managed. The clinical significance of such heteroresistance is unclear, and further study of *pncA* should be pursued.

## Introduction

Tuberculosis (TB) continues to be a major global health threat with an estimated 9.6 million new cases and 1.5 million deaths in 2014 alone [1]. Multidrug-resistant (MDR)-TB is resistant to both rifampin and isoniazid, is complicated to manage, and has poor treatment outcomes. Optimal MDR-TB therapy is guided by individualized drug susceptibility testing (DST) [2] however traditional phenotypic culture-based methods are slow. Probe-based genotypic methods have received WHO endorsement for rifampin, isoniazid, and recently for fluoroquinolones and injectable agents [3]. However these methods do not provide the specific sequence identity of the mutation, which is important to know since it is increasingly clear that particular mutations are high, moderate, or low confidence resistance mutations, or may not be associated with resistance at all [4]. Sanger sequencing of resistance determining gene regions is a popular method in well-resourced settings [5]. While sequencing capabilities are not widely available in many TB endemic countries, advances in Next-Generation Sequencing (NGS) have made this technology increasingly accessible and has been applied to the resistance determining regions of TB genes [4, 6–8]. The bulk of studies have employed whole genome sequencing (WGS) methods, however amplicon-based NGS has been promoted as a tool for future public health surveillance of MDR TB [8–11]. Therefore, in this work we performed amplicon-based NGS for 10 genes to examine the feasibility of this approach.

We were particularly interested in evaluating heteroresistance, since NGS can detect low levels of heteroresistance [12] while Sanger sequencing has less resolution [13]. Heteroresistance in TB has traditionally been detected using culture-based methods and is defined as the co-occurrence of drug-resistant and drug-sensitive colonies in the same sample. The premise of the agar proportion method is that TB drug resistance is denoted by ≥1% of colonies growing on drug-containing media [14], with a range of 1% up to 100% (fully resistant). Thus heteroresistance is traditionally defined as 1–99% resistant colonies. However with the advent of molecular testing the term is also used to describe the co-occurrence of wild-type and resistance-associated mutations in a single isolate [12, 13, 15]. This molecular heteroresistance has been described in TB for a limited number of drugs [12, 15, 16] but its prevalence and phenotypic implications across multiple drug resistance-associated genes using a sensitive NGS method is less clear. We focused this study on MDR-TB, because of the likely use of amplicon-based NGS in this scenario, plus resistant strains have revealed high rates of heteroresistance [12].

## Methods

### Samples

**Fig 1:**
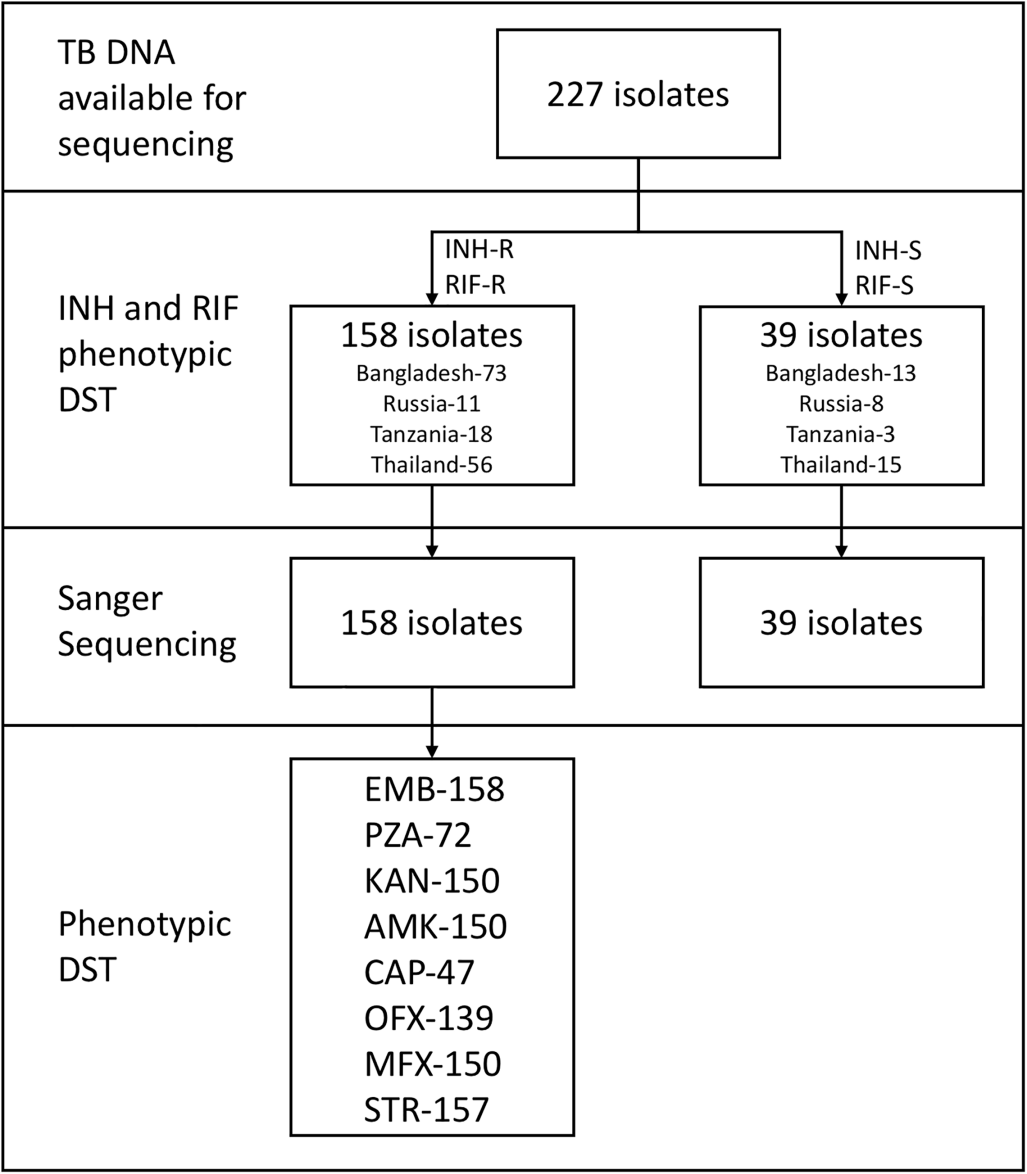
Isolate selection and description. A total of 227 tuberculosis genomic DNA extracts were available from a set of isolates that were characterized by phenotypic drug susceptibility testing. Isolate DNAs were selected for Amplicon NGS on the basis of the isolate being phenotypically characterized as MDR (INH-R RIF-R) or susceptible (INH-S RIF-S). Shown are the numbers of isolates with available data for Sanger sequencing and further phenotypic drug susceptibility testing for ethambutol (EMB), pyrazinamide (PZA), kanamycin (KAN), amikacin (AMK), capreomycin (CAP), ofloxacin (OFX), moxifloxacin (MFX), and streptomycin (STR).

The strains used in this study included *Mycobacterium tuberculosis* H37Rv (ATCC 27294) and a total of 158 clinical *M*. *tuberculosis* isolates tested as resistant to isoniazid and rifampin by phenotypic DST. *M*. *tuberculosis* isolates were obtained from four regions: consecutively obtained MDR isolates from the clinical microbiology laboratory of Siriraj Hospital, Bangkok, Thailand (n = 56); a MDR surveillance project across Dhaka, Bangladesh (n = 73); convenience samples from MDR-TB inpatients at the Kibong’oto National Tuberculosis Hospital in Tanzania (n = 18); and at the Irkutsk Regional Clinical Tuberculosis Hospital in Irkutsk, Siberia, Russian Federation (n = 11; Fig 1). We selected these isolates because all underwent phenotypic susceptibility testing using WHO endorsed methods, except that isolates from Russia were tested using the Sensititre MYCOTB plate (TREK Diagnostics, Cleveland OH, USA). Details on the phenotypic methods and critical concentrations have been previously reported [17]. Study approval was given by the University of Virginia Institutional Review Board for Health Sciences Research, the ICDDR,B Ethical Review Committee, the Tanzanian National Institute for Medical Research, and the Ethics Committee of the Scientific Centre of Family Health and Human Reproduction, Irkutsk. Written consent was obtained from all participants except for Thailand where deidentified isolates were used and therefore consent was waived by the ethics committee.

### PCR amplification of drug resistance-associated regions

For samples from Bangladesh, Tanzania, and Thailand, DNA extraction was performed by boiling as previously described [13]. For Russian samples the Boom method was used [18]. Extracts were shipped to the University of Virginia where PCR amplification, Sanger sequencing, and NGS were performed. For NGS, amplicons were generated in two stages (Fig A in S1 File). For the first stage, tailed primers (Table A in S1 File) amplified each region of interest, covering a total of 3519 nucleotides of the *M*. *tuberculosis* genome. The tails of each primer added the Read 1 and Read 2 sequencing priming sites to each amplicon. Thermocycling was performed on a BioRad myCycler or c1000 thermocycler (BioRad, Hercules, CA). The 25μL PCR cocktail included between 0.05μM and 0.1μM of each amplification primer and a commercial PCR Master Mix (5 PRIME HotMasterMix 2.5x, 5 PRIME, Hilden, Germany or Bioline MyFi, Bioline, Taunton, MA, USA), nuclease-free water, and 2μL genomic DNA. Cycling parameters were: 3 minutes at 94°C followed by 15 cycles of 95°C for 20 seconds/ 62°C for 25 seconds/ 72°C for 25 seconds, followed by a 10-minute hold at 72°C to allow for final extensions. PCR products were held at 10°C until used in the second stage PCR or stored at -20°C. First stage reactions were completed either in singleplex or multiplex. The stage 2 tailed primers used the Read 1 and Read 2 sequences as their binding sites while adding a 12-base index [19] to the 3’ area and additional sequences to complete the 5’ and 3’ adapters to make amplicons compatible with the Illumina MiSeq300 v2 kit. Each index was unique to the amplicons generated for an individual isolate. Thermocycling was also performed on the myCycler or c1000 thermocycler. All PCRs used a common “IS4” forward primer [20] and the reverse primers differed only in their index sequences. Each 25μL PCR cocktail contained 1μM each of forward and reverse primers, commercial master mix (5 PRIME HotMasterMix or BioRad iQ PowerMix, BioRad, Hercules, CA, USA), nuclease-free water, and 5μL of first stage PCR product. Cycling was programmed as 3 minutes at 95°C followed by 20 cycles of 95°C for 20 seconds, 62°C for 25 seconds, 72°C for 25 seconds, followed by a 10-minute hold at 72°C to allow for final extensions. Reactions were then stored at -20°C prior to preparation for sequencing. In addition, amplicons were generated from laboratory strain H37Rv and included in each subsequent step in the sequencing process to serve as a sequencing control.

### Sequencing on the Illumina MiSeq platform

Products amplified in the second PCR were purified using AMPure XP magnetic beads according to the manufacturer’s instructions (Beckman Coulter, Brea, CA). Purified PCR products were quantified using a Qubit Fluorimeter (Life Technologies, Carlsbad, CA) and examined for the integrity of the adaptors using SYBR Green-based PCR against PhiX control DNA (Illumina, San Diego, CA) calibration. The size distribution of amplicons was determined on an Agilent 2100 Bioanalyzer with D1000 DNA Chips. Purified libraries were pooled in an equimolar mix prior to MiSeq sequencing. Pooled libraries were first denatured in 0.1N NaOH then diluted to 8–10 pM for injection to an Illumina MiSeq300 v2 kit. Twenty percent of PhiX library was spiked to compensate for the low base complexity due to PCR amplicons. Sequencing was conducted in paired-end mode with 150 cycles for each end in addition to the 12 cycles for barcode reading. Resulting FASTQ files were collected and demultiplexed to each barcode for post run data analysis.

### Sequence quality assurance, variant calling, and analysis

The sequenced reads were first chastity filtered then assessed for quality with FastQC [21]. Ea-utils, version 1.1.2–537 [22], was then used to filter out low quality reads and trim out Illumina adaptor sequences. Reads were aligned to the H37Rv genome (GenBank NC_000962) using the Burrows-Wheeler Alignment algorithm version 0.5.7 [23], then sorted and indexed using Samblaster [24]. Variant calling was performed using FreeBayes version 0.9.14-18-g36789d8 [25]. FreeBayes parameters were set to assume a haploid organism and required a minimum alternate allele fraction of 0.05. Variants were filtered for quality and read depth, also using FreeBayes. At each genomic position (locus) we required at least 100 reads per locus for validity. The amplification and sequencing process can introduce mutations into the resulting data. This contributes to a “background” rate of mutation and was assessed by examining the sequence data generated from the H37Rv strain included in each run. To account for background, any variant at a particular locus was required to represent at least 1% of reads at that particular genomic location for an individual isolate. In instances of high background, defined as a mutation with greater than 1% of reads in H37Rv (occurred in 20/1933 loci), an isolate was only considered to be truly positive for that mutation if its level of that mutation exceeded the average level of all valid isolates (*i*.*e*. those with at least 100 reads at that particular genomic location) plus two standard deviations.

### Statistics

For quantifying NGS results when an isolate was mutant at multiple loci within a single gene, the highest percentage was counted. Receiver Operator Characteristic (ROC) and regression analyses were performed using IBM SPSS Statistics, version 24 (IBM, Armonk, NY). All P values were two-tailed.

## Results

### Prevalence of resistant mutations or heteroresistance

**Fig 2:**
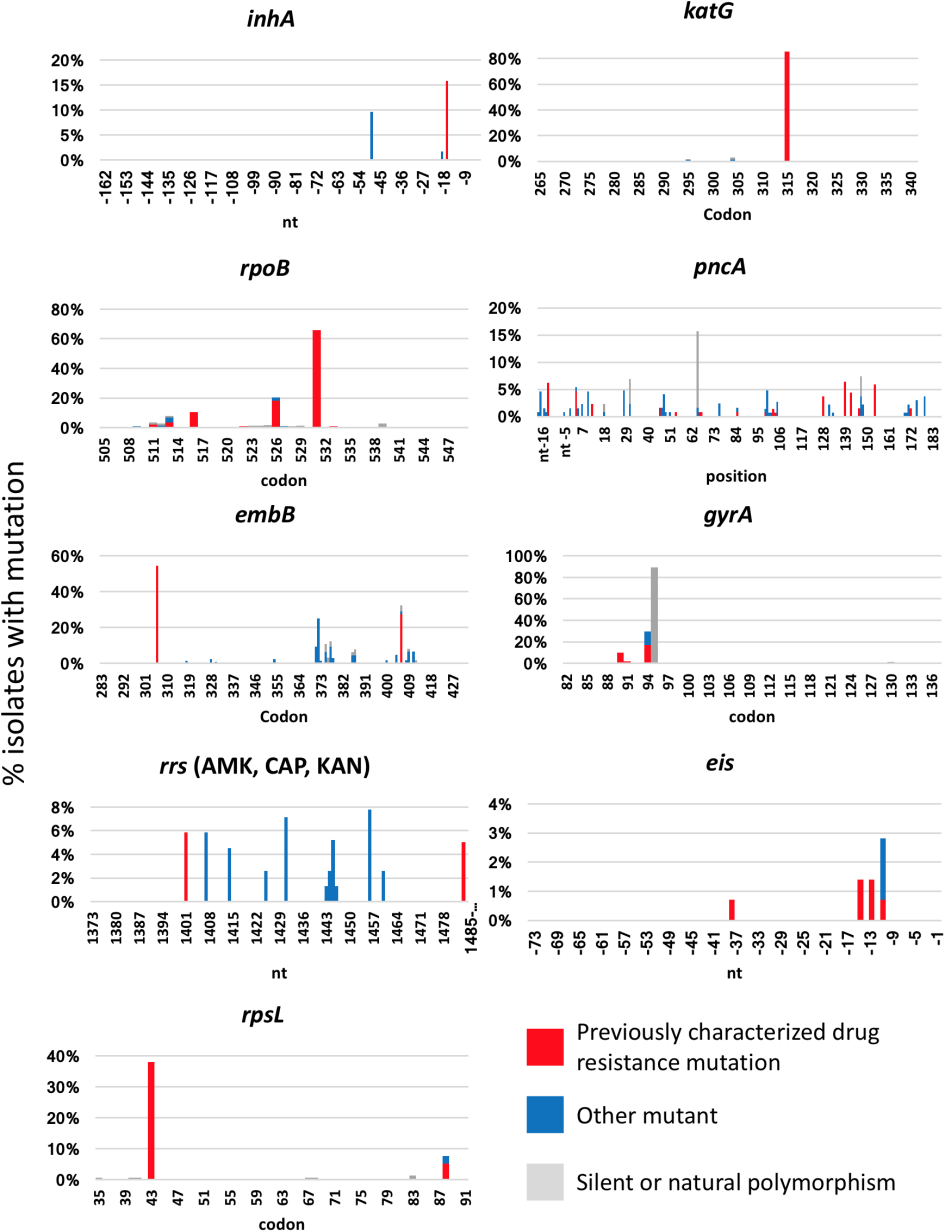
Location of mutations within *Mycobacterium tuberculosis* drug-resistance genes of 158 MDR TB isolates as detected by next generation sequencing. The y axis shows the percentage of isolates with some degree of mutation, defined as ≥1%, at each nucleotide/codon. Recognized resistance associated loci (red) were based on meta-analyses of Salamon et al. and Georghiou et al. [28, 29]. Other mutations are shown in blue, while silent mutations or natural polymorphisms are shown in grey. If, for example, a locus had resistance mutations in some isolates, and silent mutations in others, bars are stacked. Note different scales of y axes. The number of isolates with valid reads varied by locus but was at least 56 reads in all instances.

The isolates used and testing scheme are summarized in Fig 1. For all 158 MDR isolates and H37Rv, we PCR amplified the resistance determining gene regions of *inhA* and *katG* (isoniazid), *rpoB* (rifampin), *embB* (ethambutol), *gyrA* and *gyrB* (fluoroquinolones), *eis* (kanamycin), *rpsL* (streptomycin), *rrs* (one region for streptomycin and a separate region amikacin/kanamycin/capreomycin), and the entire open reading frame and promoter region of *pncA*. For each isolate this constituted 3519 nucleotides (nt), of which 321 nt were in promoter regions, 824 nt in non-coding regions and 2374 nt in open reading frames (788 codons)–see Table B in S1 File. In total this represented 1145 non-coding nucleotides and 788 codons, or 1933 total loci covering 11 total regions of 10 genes. Post-quality filtering, NGS indicated that 1811 loci were fully wild-type, whereas 122 (6.3%) exhibited at least some mutant population (≥1% of reads) in at least one isolate. Locations of these mutant loci were dispersed across all genes (Fig 2). Thirty-one of the 122 mutant loci included silent mutations or the *gyrA*95 natural polymorphism [26], and 46 of the loci included accepted drug resistance-associated mutations according to the ReSeqTB online database [27] and meta-analyses from Salamon et al. and Georghiou et al. [28, 29]. The remainder were other mutations of unclear significance. Not surprisingly in these MDR-TB strains, a large number of isolates had mutation at *katG* 315 and the common *rpoB* 531 mutations, and the *pncA* mutations were scattered throughout the gene. Of the 35 MDR-TB isolates that had sufficient valid reads at all 46 recognized resistance-associated loci, and excluding completely (>99%) wild-type or mutant loci, 15 (43%) had no heteroresistance in any loci, 7 (20%) had one heteroresistant locus, and 13 (37%) had two or more heteroresistant loci (Fig B in S1 File). As a control, H37Rv was also subjected to amplicon NGS and was 100% wild-type at all loci as described in the methods.

### Extent of heteroresistance

**Fig 3:**
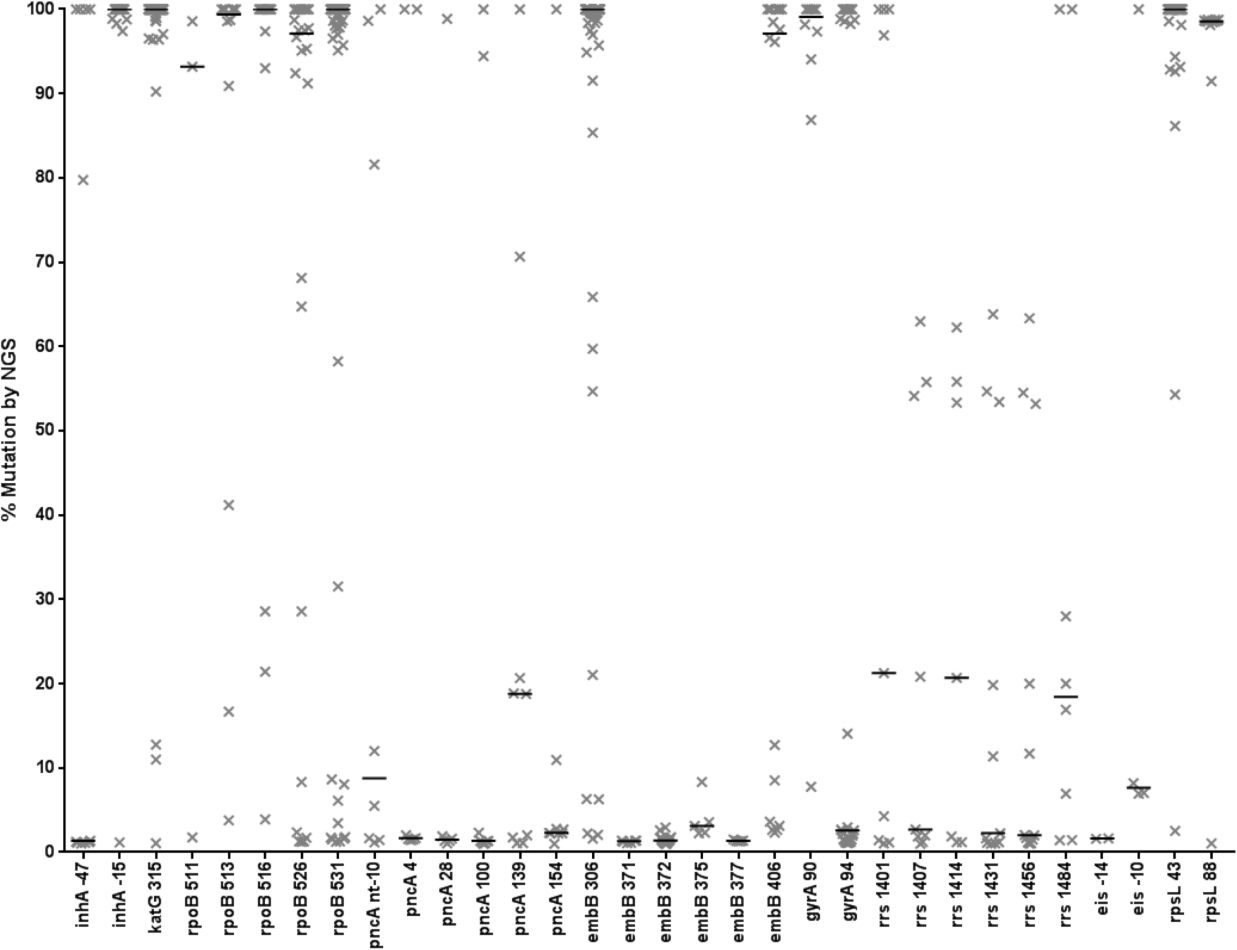
Degree of heteroresistance at major loci. Loci whereby at least 5% of isolates had some degree of mutation are shown, as well as known *rpoB* and *eis* mutations. The y-axis shows the degree of heteroresistance, with each ‘X’ symbol representing a single isolate. Horizontal lines are median values. Loci are shown on the x axis. In rare instances whereby an isolate had more than one mutation at the same locus, the mutation with the highest degree of heteroresistance is shown.

With the prevalence of heteroresistance established, we then examined the degree of heteroresistance within these isolates. For certain loci such as *gyrA*94 and *pncA*-4/154, the median degree of mutation in these isolates was <10% (Fig 3). Conversely, for other loci such as *gyrA*90, *inhA*-15, *katG*315, *rpoB*513/531/516/526/531, *embB*306/406, and *rpsL*43/88, the median mutation level was >90%. Intermediate levels (e.g. degree of mutation between 10–90%) were less common, and was noted in only 51 of the 284 (18%) heteroresistant instances shown in Fig 3.

### Comparison of NGS to Sanger sequencing and phenotypic susceptibility testing

**Fig 4:**
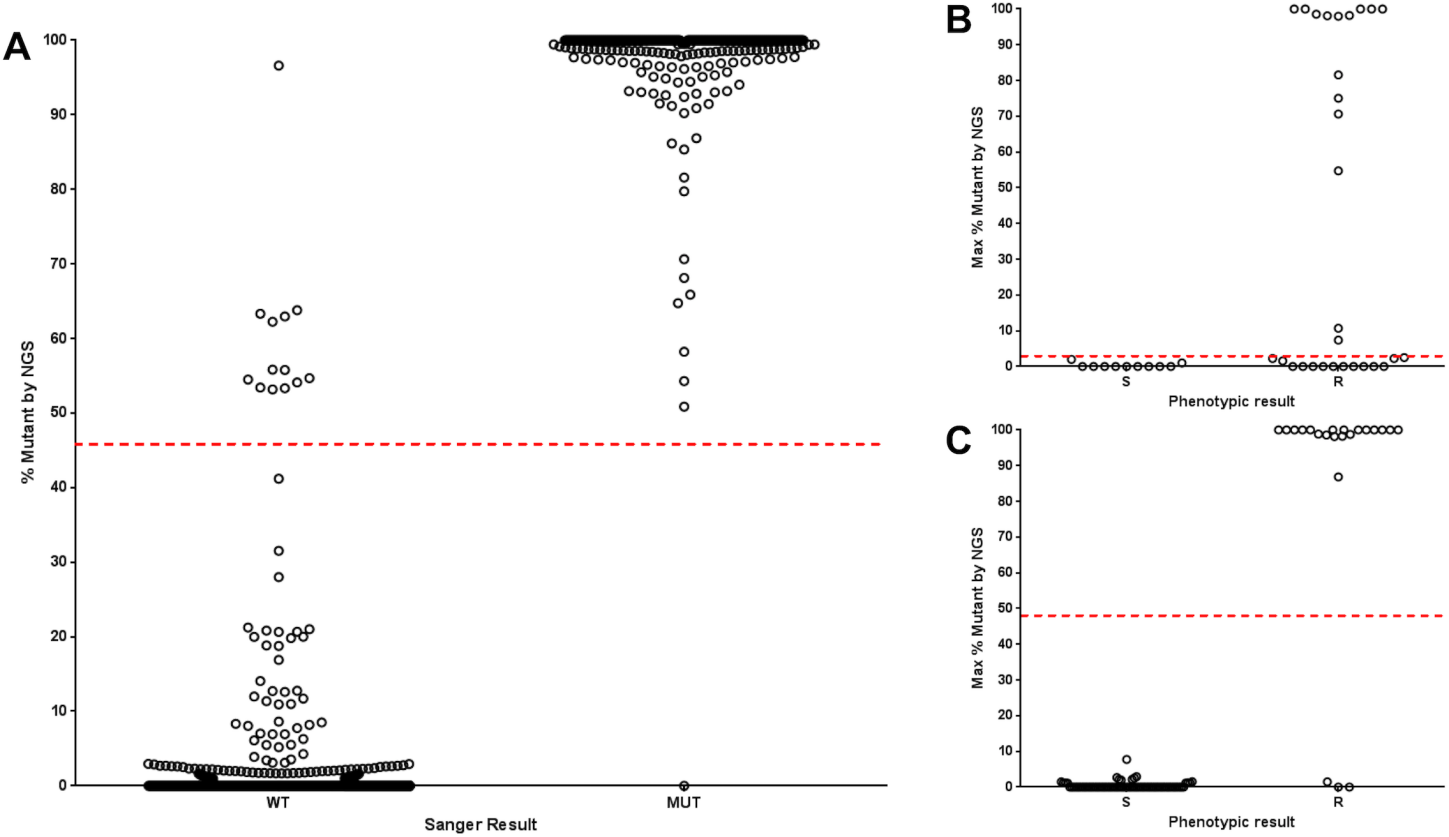
Degree of heteroresistance by NGS compared with Sanger sequencing result. (A) All isolates and loci from Fig 2 are shown. The dashed line shows the ROC heteroresistance breakpoint (46.1%) that correlates optimally with the Sanger result, yielding an accuracy of x/y (99%). (B) *pncA* resistance associated mutations are shown, and the dashed line shows the ROC optimized heteroresistant breakpoint (2%) to correlate with the phenotypic result. (C) *gyrA* resistance associated mutations are shown, and the dashed line shows the ROC optimized heteroresistant breakpoint (49%) to correlate with the phenotypic result.

Comparisons between the NGS and Sanger sequencing results are shown in Table 1 (see also S1 NGS summary MDR). Heteroresistance was detected by NGS in 5.2% (187/3586) of instances that were called wild type by Sanger sequencing, and in 21.2% (96/453) of instances that were called mutant by Sanger sequencing. As controls, we evaluated 39 isoniazid- and rifampin-susceptible isolates from the same repositories (see S2 NGS summary Susceptibles), and the average prevalence of heteroresistance in the Table 1 loci was not statistically different (paired t-test P > 0.05; data not shown). We next compared the degree of heteroresistance versus the Sanger result (Fig 4A). ROC analysis revealed that a 46.1% level of mutation (or 53.9% wild type) was the breakpoint that optimized NGS accuracy versus Sanger. Of note, there were very few misidentified nucleotide discrepancies between Sanger and NGS (2/4039). We next compared the NGS results against phenotypic susceptibility testing (Table C in S1 File). First, in this repository, the accuracy of Sanger detection of recognized resistance mutations versus phenotypic resistance ranged from 63% for pyrazinamide to 100% for amikacin and capreomycin (average 86% ±16 for the 8 different drugs). We hypothesized that the sensitivity to detect resistance would be increased by the ability of NGS to detect minor populations of mutants. However, generally, increases in sensitivity were offset by decreases in specificity, such that comparing NGS detection of any mutation (defined as >1%) versus phenotypic susceptibility results reduced accuracy to an average of 82%±11 for all 8 drugs. There were individual differences by drug, however, such that a ROC optimized 2% heteroresistant breakpoint for *pncA* mutations did appear to improve the sensitivity for detecting pyrazinamide resistance versus Sanger without a specificity cost (accuracy 73% by NGS versus 63% by Sanger, however 95% confidence intervals overlap; Fig 4B). For *gyrA*, by contrast, the optimized breakpoint was 47% (Fig 4C), a point serendipitously approximated by Sanger.

**Table 1:** Number of MDR TB isolates with wild-type, mutant, and heteroresistant loci as revealed by Sanger and NGS.

Locus	Sanger WT/ NGS 100% WT	Sanger WT/ NGS heteroresistant	Sanger mutant/ NGS heteroresistant*	Sanger mutant/ NGS 100% MUT	Frequency of heteroresistance by NGS
inhA -47	102	6	1	4	6%
inhA -15	95	1	4	13	4%
katG 315	20	3	9	104	9%
rpoB 511	139	1	2	0	2%
rpoB 513	132	1	3	6	3%
rpoB 516	127	1	3	11	3%
rpoB 526	113	5	11	13	11%
rpoB 531	49	11	11	71	15%
pncA nt -11	120	5	2	1	5%
pncA 4	121	5	0	2	4%
pncA 28	118	5	1	0	5%
pncA 100	137	6	1	1	5%
pncA 139	131	7	1	1	6%
pncA 154	127	7	0	1	5%
embB 306	59	6	15	55	16%
embB 371	57	6	0	0	10%
embB 372	47	16	0	0	25%
embB 375	58	5	0	0	8%
embB 377	58	5	0	0	8%
embB 406	43	8	3	7	18%
gyrA 90	90	1	4	5	5%
gyrA 94	70	19	4	7	23%
rrs 1401	144	5	1	3	4%
rrs 1407	144	9	0	0	6%
rrs 1414	146	7	0	0	5%
rrs 1431	142	11	0	0	7%
rrs 1456	141	12	0	0	8%
rrs 1484	150	6	0	2	4%
eis -14	139	2	0	0	1%
eis -10	138	3	0	0	2%
rpsL 43	97	1	9	50	6%
rpsL 88	145	1	11	0	8%
**TOTALS**	**3399**	**187**	**96**	**357**	**Mean 8±6%**

All genes from Fig 3 are shown. Loci with previously characterized resistance-associated mutations (from references 26–28) are underlined, other mutations are not.

*There were just 2 instances of Sanger mutant/NGS 100% wild-type and 0 instances of Sanger WT/NGS 100% mutant.

**Fig 5:**
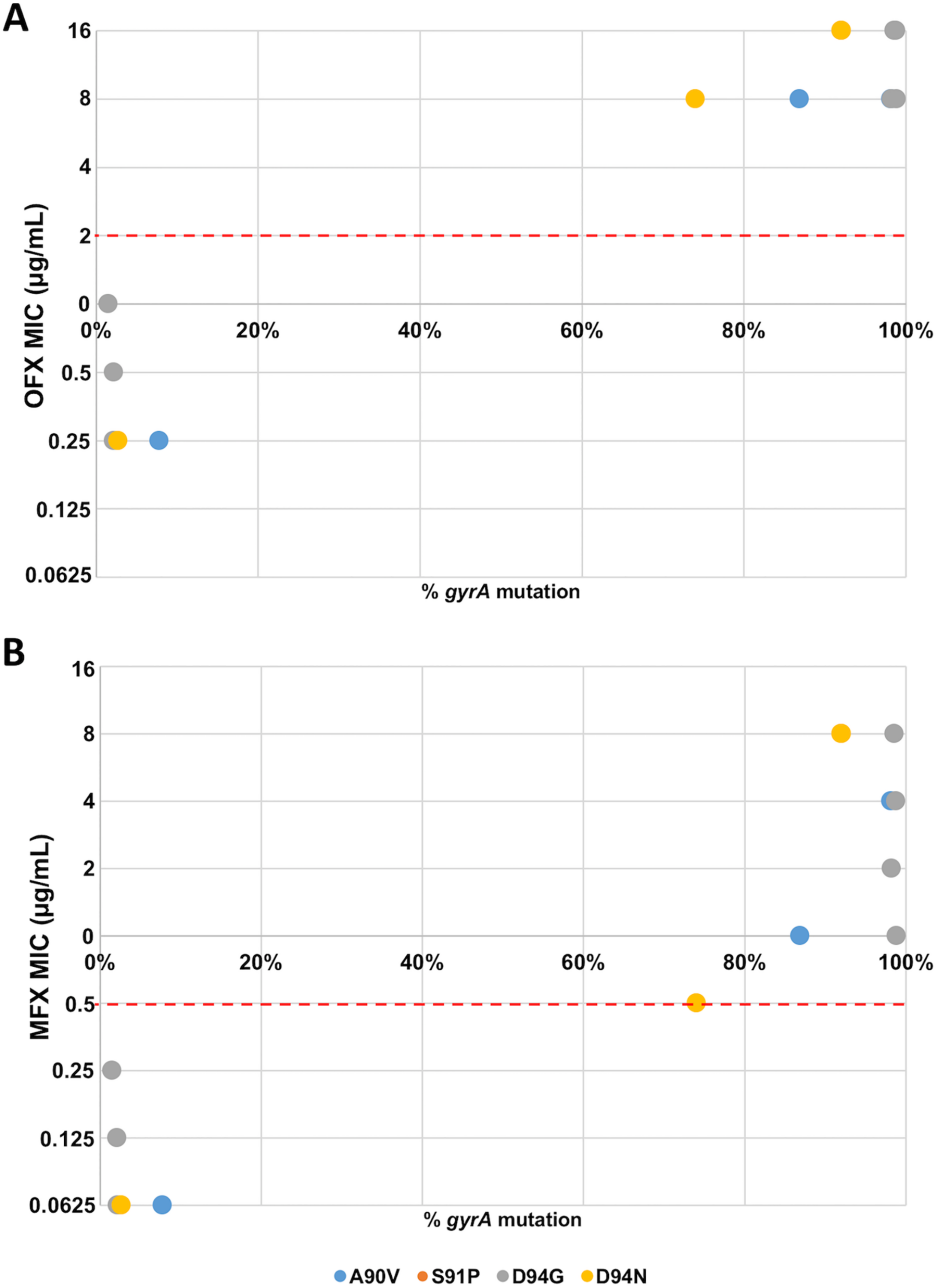
Heteroresistance in *gyrA* versus minimal inhibitory concentration. Each dot represents a single isolate with heteroresistance (1–99%) in *gyrA* (n = 14). The minimum inhibitory concentration cutoff for tests performed by TREK Sensititre Mycobacteria plate is shown as a dashed red line. The same trends are seen if we include all available isolates (n = 75) including those with 0% and 100% mutation.

MIC results were available for the majority of isolates for streptomycin, ethambutol, kanamycin, amikacin, capreomycin, ofloxacin, and moxifloxacin. We compared the MIC and the degree of heteroresistance for the fluoroquinolones and ethambutol. This analysis was not possible for streptomycin because there was minimal heteroresistance, nor for kanamycin, amikacin, and capreomycin because there was minimal phenotypic resistance. In examining the relationship between degree of mutation and phenotypic resistance to ofloxacin and moxifloxacin there were two clusters of isolates, those with high degrees of *gyrA* mutation and phenotypic resistance and those with low degrees of mutation and phenotypic resistance (Fig 5). By contrast, there was no relationship between heteroresistance in *embB* resistance-associated mutations and ethambutol resistance (Fig C in S1 File).

## Discussion

In this work we employed NGS to examine heteroresistance in 1933 genotypic loci of 158 MDR-TB strains and 39 drug susceptible TB controls. We found heteroresistance to be an unexpectedly common phenomenon. Not including isolates that were completely (>99%) wild-type or mutant at certain loci, we documented that heteroresistance occurs at least once in about half of strains in these important resistance-determining regions. Therefore for future applications of NGS for TB drug susceptibility testing, heteroresistance and quantities of heteroresistance will need to be bioinformatically managed and clinical implications better understood.

We hypothesized that NGS’s sensitivity to detect heteroresistance might improve the sensitivity of genotypic methods for DST, but with the possible exception of pyrazinamide this was not the case in these isolates. Surprising to us, low heteroresistant *gyrA* mutants were still phenotypically susceptible and had low MIC. We did see a possible advantage of NGS over Sanger for providing a pyrazinamide susceptibility result, where using a heteroresistance breakpoint of 2% gave an accuracy of 73% whereas Sanger yielded an accuracy of only 62%. These gains were seen in improved sensitivity for detecting pyrazinamide resistance, though there still remained a number of phenotypically pyrazinamide resistant strains that were fully (100%) wild-type in all *pncA* loci. A similarly small improvement of pyrazinamide susceptibility accuracy with NGS versus Sanger has been noted by others [30]. Further study with larger numbers of isolates is needed. Certainly PZA is a particularly important drug susceptibility result, since PZA is an important component of current and future TB regimens, both for drug susceptible and drug resistant TB. Moreover, the clinical significance of this common phenomena of heteroresistance, often not detected by Sanger or phenotypic methods, is unclear.

We found the amplicon-based NGS method quite cumbersome to analyze. In our hands, after quality filtering of raw sequence, alignment to the reference, and variant calling, all other data management was performed manually without the aid of a bioinformatic pipeline. This included the parsing of ORF variants into codons, calculation of variant vs. wild-type frequencies, and applying quality filtering to the translated data. There is a paucity of published bioinformatic pipelines built for the analysis of amplicon-based NGS data from *M*. *tuberculosis* [31], compared with several pipelines for whole genome approaches [32–35]. With either amplicon NGS or WGS, heteroresistance will clearly arise and will need to be bioinformatically managed. Based on our results we would suggest that a heteroresistance breakpoint of ~50% in *gyrA* would optimize the fluoroquinolone susceptibility result, whereas a lower breakpoint may be advantageous for *pncA*. Certainly validating these breakpoints in larger repositories will be important, as will fortification of databases that identify whether particular mutations are high, moderate, or low confidence resistance mutations, or not resistance associated at all.

There were limitations to this work. The degree of heteroresistance seen in this study may be underestimated by the fact that we performed PCR amplification before sequencing, as this will preferentially amplify the major populations. This may explain why intermediate levels of heteroresistance were rarely observed. It would be useful to compare a whole genome approach (without amplification). Such an approach would help clarify if heteroresistance is concentrated in resistance-associated genes versus other parts of the genome, in other words whether such heteroresistance occurs via selection. However, whole genome approaches would reduce sequencing depth in our regions of interest. As it was, the number of valid reads for particular isolate loci was often limited. Additionally, the amplicon NGS approach cannot determine whether heteroresistance occurred because of co-infection with two different strains, or by acquired resistance. The DNA extraction methodology and phenotypic DST methodology were not identical at all sites, which could impact the results. Finally, the MDR strains were from a range of geographies and there were a small number of strains resistant to second-line drugs, limiting power.

In summary, NGS of amplicons of key TB drug resistance determining regions is feasible but in our hands is procedurally and bioinformatically complex. Heteroresistance is common, will need to be addressed in future applications, particularly since quantitative heteroresistance information may yield clinically relevant drug susceptibility information for fluoroquinolones and pyrazinamide.

## Supporting information

S1 File **Fig A. Fig Amplicon generation.** Amplicons were generated as shown. The first stage amplifies the region of interest from genomic DNA while simultaneously adding the sequencing priming sites (“Read 1” and “Read 2” priming sites). The second stage adds a 12bp index and adapter sequences (“IS4” and “End Adapter”) to make the amplicon compatible with Illumina sequencing chemistry. Each index is unique to the amplicons for an individual isolate, however IS4 and End Adapter sequences are the same for all amplicons; **Table A. Amplification primers used in this study**; **Table B. NGS coverage of resistance-associated genes**; **Fig B. Frequency of heteroresistance across all genes tested.** All 158 MDR TB isolates underwent NGS of the 11 gene amplicons, of which 35 had sufficient read depth at all 46 recognized drug-resistance associated loci. The number of isolates (y axis) with heteroresistant instances (x axis) is shown; **Table C. Correlation of NGS and Sanger with Phenotypic Drug Susceptibility Testing**; **Fig C. Heteroresistance in *embB* versus MIC.** Each dot represents a single isolate (multiple isolates may be overlaid) in a recognized resistance-associated mutation based on the meta-analysis of Salamon et al. A total of 66 isolates are shown. The percent *embB* mutation is shown on the x-axis while the ethambutol minimum inhibitory concentration is shown on the y-axis. The minimum inhibitory concentration cutoff is shown as a dashed red line.
(DOCX)

S1 NGS Summary MDR.xls. A combined summary of the phenotypic, Sanger, and Amplicon-Next Generation Sequencing results of the 158 MDR (isoniazid resistant/rifampin resistant) strains included in this study.
(XLSX)

S2 NGS Summary Susceptibles.xls. A combined summary of the phenotypic, Sanger, and Amplicon-Next Generation Sequencing results of the 39 susceptible (isoniazid sensitive/rifampin sensitive) strains used in this study.
(XLSX)
