# Correction: Prevalence and extent of heteroresistance by next generation sequencing of multidrug-resistant tuberculosis

**DOI:** 10.1371/journal.pone.0181284

**Published:** 2017-07-07

**Authors:** Darwin J. Operario, Alexander F. Koeppel, Stephen D. Turner, Yongde Bao, Suporn Pholwat, Sayera Banu, Suporn Foongladda, Stellah Mpagama, Jean Gratz, Oleg Ogarkov, Svetlana Zhadova, Scott K. Heysell, Eric R. Houpt

The eleventh author’s name is spelled incorrectly. The correct name is: Svetlana Zhdanova.
