# Supplementary material for: Prevalence and extent of heteroresistance by next generation sequencing of multidrug-resistant tuberculosis
Source: PLoS One. 2017 May 18;12(5):e0176522. doi: 10.1371/journal.pone.0176522 (PMC5436647; doi:10.1371/journal.pone.0176522)

**Figure A**


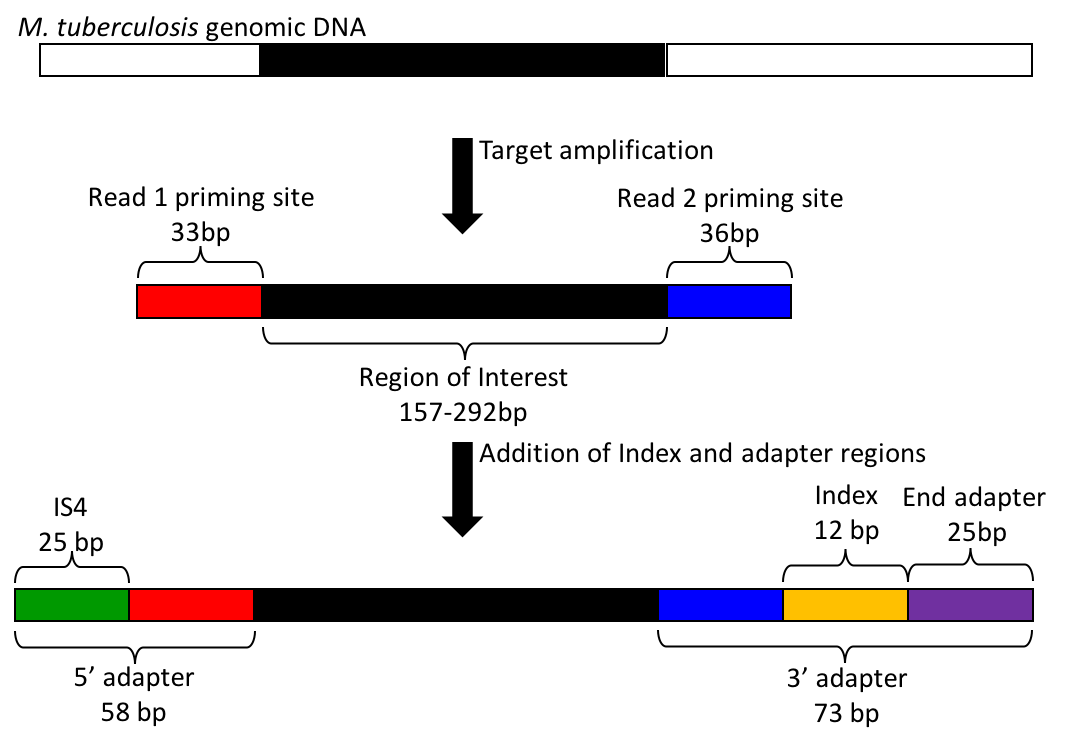


**Table A**

| **Oligonucleotide name** | **Sequence, 5’🡪3’** |
| --- | --- |
| illumina_inhA_F | ACACTCTTTCCCTACACGACGCTCTTCCGATCTTGCCCAGAAAGGGATCCGTCATG |
| illumina_katG_F | ACACTCTTTCCCTACACGACGCTCTTCCGATCTAACGACGTCGAAACAGCGG |
| illumina_rpoB_F | ACACTCTTTCCCTACACGACGCTCTTCCGATCTGTCGCCGCGATCAAGGAG |
| illumina_gyrA_F | ACACTCTTTCCCTACACGACGCTCTTCCGATCTCGGTTGCCGAGACCATG |
| illumina_embB_amp1_F | ACACTCTTTCCCTACACGACGCTCTTCCGATCTCTGACCGACGCCGTGG |
| illumina_embB_amp2_F | ACACTCTTTCCCTACACGACGCTCTTCCGATCTGGCCGCCGGGCTAGTG |
| illumina_rrs_reg1_amp1_F | ACACTCTTTCCCTACACGACGCTCTTCCGATCTCGGGTTCTCTCGGATTGACG |
| illumina_rrs_reg1_amp2_F | ACACTCTTTCCCTACACGACGCTCTTCCGATCTGCTTAACTGTGAGCGTGC |
| illumina_rrs_reg1_amp3_F | ACACTCTTTCCCTACACGACGCTCTTCCGATCTGCAGTAACTGACGCTGAGG |
| illumina_rrs_reg2_amp1_F | ACACTCTTTCCCTACACGACGCTCTTCCGATCTCAACGCTGCGGTGAATACG |
| illumina_rrs_reg2_amp2_F | ACACTCTTTCCCTACACGACGCTCTTCCGATCTAAGGTGGGATCGGCGATT |
| illumina_eis_F | ACACTCTTTCCCTACACGACGCTCTTCCGATCTTGATCCTTTGCCAGACACTG |
| illumina_rpsL_F | ACACTCTTTCCCTACACGACGCTCTTCCGATCTGCAGCGTCGTGGTGTATG |
| illumina_pncA_amp1_F | ACACTCTTTCCCTACACGACGCTCTTCCGATCTGGCGTCATGGACCCTATATC |
| illumina_pncA_amp2_F | ACACTCTTTCCCTACACGACGCTCTTCCGATCTGCCATCAGCGACTACCTG |
| illumina_pncA_amp3_F | ACACTCTTTCCCTACACGACGCTCTTCCGATCTGAGGCGGTGTTCTACAAGG |
| illumina_gyrB_F | ACACTCTTTCCCTACACGACGCTCTTCCGATCTCGTAAGGCACGAGAGTTGGT |
| illumina_inhA_R | GTGACTGGAGTTCAGACGTGTGCTCTTCCGATCTCCTCCGGTAACCAGGACTGAAC |
| illumina_katG_R | GTGACTGGAGTTCAGACGTGTGCTCTTCCGATCTCCCAGGGCTCTTCGTCAGCTC |
| illumina_rpoB_R | GTGACTGGAGTTCAGACGTGTGCTCTTCCGATCTCCGTAGTGCGACGGGTGCA |
| illumina_gyrA_R | GTGACTGGAGTTCAGACGTGTGCTCTTCCGATCTCCTTTCCCTCAGCATCTCCATC |
| illumina_embB_amp1_R | GTGACTGGAGTTCAGACGTGTGCTCTTCCGATCTCCTCACGCGACAGCAGCAG |
| illumina_embB_amp2_R | GTGACTGGAGTTCAGACGTGTGCTCTTCCGATCTCCGAATGCGGCGGTAACGACG |
| illumina_rrs_reg1_amp1_R | GTGACTGGAGTTCAGACGTGTGCTCTTCCGATCTCCTCTAGTCTGCCCGTATCG |
| illumina_rrs_reg1_amp2_R | GTGACTGGAGTTCAGACGTGTGCTCTTCCGATCTCCGTATCTAATCCTGTTCGCTCC |
| illumina_rrs_reg1_amp3_R | GTGACTGGAGTTCAGACGTGTGCTCTTCCGATCTCCCATCGAATTAATCCACATGCTC |
| illumina_rrs_reg2_amp1_R | GTGACTGGAGTTCAGACGTGTGCTCTTCCGATCTCCCCGGTACGGCTACCTTGTTA |
| illumina_rrs_reg2_amp2_R | GTGACTGGAGTTCAGACGTGTGCTCTTCCGATCTCCGTCCGAGTGTTGCCTCAGG |
| illumina_eis_R | GTGACTGGAGTTCAGACGTGTGCTCTTCCGATCTCCGATCCATGTACAGCGCCATC |
| illumina_rpsL_R | GTGACTGGAGTTCAGACGTGTGCTCTTCCGATCTCCATGATCTTGTAGCGCACACC |
| illumina_pncA_amp1_R | GTGACTGGAGTTCAGACGTGTGCTCTTCCGATCTCCATGGTAGTCCGCCGCTTC |
| illumina_pncA_amp2_R | GTGACTGGAGTTCAGACGTGTGCTCTTCCGATCTCCCCGTTCTCGTCGACTCCTTC |
| illumina_pncA_amp3_R | GTGACTGGAGTTCAGACGTGTGCTCTTCCGATCTCCATCAGGAGCTGCAAACCAAC |
| illumina_gyrB_R | GTGACTGGAGTTCAGACGTGTGCTCTTCCGATCTCCCAGCGCCGTGATGATCGCC |
| IS4 primer | AATGATACGGCGACCACCGAGATCTACACTCTTTCCCTACACGACGCTCTTCCGATCT |
| PCR Stage 2 reverse primer* | GGAGATCGGAAGAGCACACGTCTGAACTCCAGTCACNNNNNNNNNNNNTATCTCGTAT  GCCGTCTTCTGCTTG |

*Each Stage 2 reverse primer contains a 12nt index sequence (“barcode”) which was unique for each isolate, represented by a series of ‘N’s in the table above.

**Table B**

| **Gene** | **Covered Length (nt)** | **Amplicon Count** | **Promoter (nt)** | **ORF (codon number)** | **Non-coding sequence** |
| --- | --- | --- | --- | --- | --- |
| *inhA* | 162 | 1 | -162 to -1 | n/a | n/a |
| *katG* | 271 | 1 | n/a | 258-347 (+1nt) | n/a |
| *rpoB* | 174 | 1 | n/a | 498 to 555 (*E. coli* numbering) | n/a |
| *pncA* | 642 | 3 | -80 to -1 | 1-187 (+1nt) | n/a |
| *embB* | 489 | 2 | n/a | 278 to 439 | n/a |
| *gyrA* | 207 | 1 | n/a | (2nt),77 to 144,(+1nt) | n/a |
| *gyrB* | 266 | 1 | n/a | 421 to 508 (+2nt) | n/a |
| *rrs*-STR | 504 | 3 | n/a | n/a | nt456 to nt959 |
| *rrs*-injectables | 320 | 2 | n/a | n/a | nt1354 to nt1673 |
| *eis* | 274 | 1 | -79 to -1 | 1-67 | n/a |
| *rpsL* | 210 | 1 | n/a | (1nt), 29 to 97, (+2nt) | n/a |
| **Totals** | **3519** | **17** | **321nt** | **788 codons**  **+10nt** | **824nt** |

**Figure B**

**Table C**

| Drug | Gene Targets(s) | Sanger result | Phenotypic Result | | % Accuracy, Sanger | Raw NGS Result (MUT= ≥1% mutant) | Phenotypic Result | | % Accuracy, raw NGS | % mutation break point from ROC analysis | NGS result, post-break point | Phenotypic Result | | % Accuracy, break point applied |
| --- | --- | --- | --- | --- | --- | --- | --- | --- | --- | --- | --- | --- | --- | --- |
|  |  |  | R | S |  |  | R | S |  |  |  | R | S |  |
| EMB | *embB* | MUT | 61 | 22 | 70 | MUT | 61 | 22 | 70 | ≥98=MUT | MUT | 54 | 15 | 70 |
|  |  | WT | 10 | 13 |  | WT | 10 | 13 |  |  | WT | 17 | 20 |  |
| PZA | *pncA* | MUT | 14 | 0 | 63 | MUT | 19 | 2 | 70 | ≥2=MUT | MUT | 18 | 0 | 73 |
|  |  | WT | 15 | 11 |  | WT | 10 | 9 |  |  | WT | 11 | 11 |  |
| KAN | *eis-rrs* | MUT | 6 | 1 | 97 | MUT | 8 | 9 | 93 | ≥2=MUT | MUT | 8 | 6 | 95 |
|  |  | WT | 3 | 123 |  | WT | 1 | 115 |  |  | WT | 1 | 118 |  |
| AMK | *rrs* | MUT | 5 | 0 | 100 | MUT | 5 | 10 | 93 | ≥63=MUT | MUT | 5 | 0 | 100 |
|  |  | WT | 0 | 146 |  | WT | 0 | 136 |  |  | WT | 0 | 146 |  |
| CAP | *rrs* | MUT | 3 | 0 | 100 | MUT | 3 | 2 | 96 | ≥64=MUT | MUT | 3 | 0 | 100 |
|  |  | WT | 0 | 45 |  | WT | 0 | 43 |  |  | WT | 0 | 45 |  |
| OFX | *gyrA* | MUT | 18 | 0 | 96 | MUT | 20 | 15 | 82 | ≥47=MUT | MUT | 19 | 0 | 97 |
|  |  | WT | 4 | 70 |  | WT | 2 | 55 |  |  | WT | 3 | 70 |  |
| MFX | *gyrA* | MUT | 16 | 3 | 94 | MUT | 18 | 18 | 80 | ≥47=MUT | MUT | 17 | 3 | 95 |
|  |  | WT | 3 | 72 |  | WT | 1 | 57 |  |  | WT | 2 | 72 |  |
| STR | *rpsL* | MUT | 65 | 5 | 69 | MUT | 65 | 5 | 69 | ≥92=MUT | MUT | 63 | 3 | 69 |
|  |  | WT | 44 | 43 |  | WT | 44 | 43 |  |  | WT | 46 | 45 |  |
| OVERALL |  |  |  |  | 86 |  |  |  | 82 |  |  |  |  | 87 |

**Figure C**


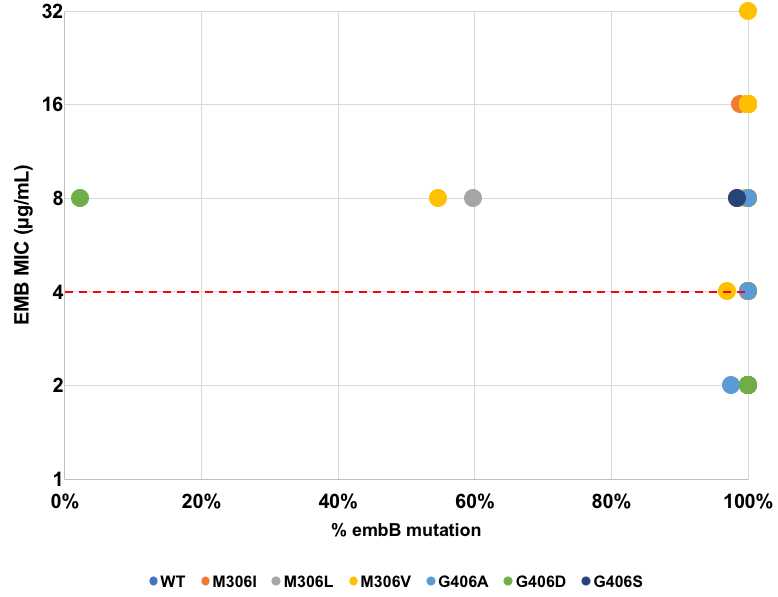

Supplement: S1 File — Fig A. Fig Amplicon generation. Amplicons were generated as shown. The first stage amplifies the region of interest from genomic DNA while simultaneously adding the sequencing priming sites (“Read 1” and “Read 2” priming sites). The second stage adds a 12bp index and adapter sequences (“IS4” and “End Adapter”) to make the amplicon compatible with Illumina sequencing chemistry. Each index is unique to the amplicons for an individual isolate, however IS4 and End Adapter sequences are the same for all amplicons; Table A. Amplification primers used in this study; Table B. NGS coverage of resistance-associated genes; Fig B. Frequency of heteroresistance across all genes tested. All 158 MDR TB isolates underwent NGS of the 11 gene amplicons, of which 35 had sufficient read depth at all 46 recognized drug-resistance associated loci. The number of isolates (y axis) with heteroresistant instances (x axis) is shown; Table C. Correlation of NGS and Sanger with Phenotypic Drug Susceptibility Testing; Fig C. Heteroresistance in embB versus MIC. Each dot represents a single isolate (multiple isolates may be overlaid) in a recognized resistance-associated mutation based on the meta-analysis of Salamon et al. A total of 66 isolates are shown. The percent embB mutation is shown on the x-axis while the ethambutol minimum inhibitory concentration is shown on the y-axis. The minimum inhibitory concentration cutoff is shown as a dashed red line. (DOCX) [file pone.0176522.s001.docx]
